# Polybutylene succinate artificial scaffold for peripheral nerve regeneration

**DOI:** 10.1002/jbm.b.34896

**Published:** 2021-06-27

**Authors:** Luca Cicero, Mariano Licciardi, Roberta Cirincione, Roberto Puleio, Gaetano Giammona, Giuseppe Giglia, Pierangelo Sardo, Giulio Edoardo Vigni, Alessio Cioffi, Antonino Sanfilippo, Giovanni Cassata

**Affiliations:** ^1^ Centro Mediterraneo Ricerca e Training (Ce.Me.Ri.T) Istituto Zooprofilattico Sperimentale della Sicilia “A. Mirri” Palermo Italy; ^2^ Dipartimento di Scienze e Tecnologie Biologiche Chimiche e Farmaceutiche (STEBICEF) Università degli Studi di Palermo Palermo Italy; ^3^ Dipartimento di Biomedicina Neuroscienze e Diagnostica Avanzata (BiND) Università degli Studi di Palermo Palermo Italy; ^4^ Dipartimento di Discipline Chirurgiche, Oncologiche e Stomatologiche Università degli Studi di Palermo Palermo Italy

**Keywords:** artificial conduits, electrospinning, nerve regeneration, poly(1,4‐butylene succinate) (PBS), sciatic nerve

## Abstract

Regeneration and recovery of nerve tissues are a great challenge for medicine, and positively affect the quality of life of patients. The development of tissue engineering offers a new approach to the problem with the creation of multifunctional artificial scaffolds that act on various levels in the damaged tissue, providing physical and biochemical support for the growth of nerve cells. In this study, the effects of the use of a tubular scaffold made of polybutylene succinate (PBS), surgically positioned at the level of a sciatic nerve injured in rat, between the proximal stump and the distal one, was investigated. Scaffolds characterization was carried out by scanning electron microscopy and X‐ray microcomputed tomography and magnetic resonance imaging, in vivo. The demonstration of the nerve regeneration was based on the evaluation of electroneurography, measuring the weight of gastrocnemius and tibialis anterior muscles, histological examination of regenerated nerves and observing the recovery of the locomotor activity of animals. The PBS tubular scaffold minimized iatrogenic trauma on the nerve, acting as a directional guide for the regenerating fibers by conveying them toward the distal stump. In this context, neurotrophic and neurotropic factors may accumulate and perform their functions, while invasion by macrophages and scar tissue is hampered.

## 1 INTRODUCTION

Damage to the central and peripheral nervous system causes irreversible effects and current treatment strategies do not offer reliable results. In particular, peripheral nerve injuries (PNI) include a wide range of disorders in neurologic and neurosurgical practice, and they are still today a serious medical and public health problem.^1^ Diseases involving the peripheral nervous system, particularly in the younger population, often originate from motor vehicle accidents or high velocity trauma, leading to life‐long disabling neurologic dysfunction and devastating impacts on patients' daily functions and routines. Up to 33% of all PNI shows incomplete nerve recovery and poor functional outcomes, resulting in motor and sensory disabilities, neuropathic chronic pain, end target muscle atrophy and profound weakness.^2^ Despite noticeable advancements in instrumentation and microsurgical techniques, long‐term prognosis in patients with severe nerve lesions and extended axonal degeneration remains discouraging.^3^ Therefore, it is often difficult to achieve complete peripheral neural regeneration (rejoin the nerve gaps) and to restore function of target nerve‐related muscles.^4^

A nerve gap is defined as the distance between two ends of a severed nerve, resulting from nerve retraction or loss of tissue from injury.^5^ Management of nerve gaps depends on the length of the nerve defect, the nerve diameter, the availability of the proximal stump and the proximal or distal site of the lesion. Different types of surgical therapeutic approaches are commonly used for sensory and motor functional recovery following PNIs.^6^

With nerve gaps less than 1 cm, in the absence of tension between the ends of the severed nerve, the gold‐standard method for treatment of nerve damage is the direct nerve repair with microsurgical techniques. This approach hopes to provide continuity between the distal and the proximal part of the transected nerves.^7^ In particular, epineural repair provides for achieving the tension‐free natural connection of the nerve tissue and accurate alignment of the nerve fascicles. In this case, a rapid functional recovery is possible, especially if the denervation time is less than 6 months and the age of the patient is less than 50 years.^8^, ^9^

In the past, fibrin glue has been utilized for primary sutureless nerve cooptation by using an adhesive material known as fibrin sealants.^10^ In clinical practice, it is considered as an efficient technique, quick and easy to use, as it ensures versatility for different nerve repair situations, a shorter recovery time and no‐induction of inflammation or fibrosis.^11^

Recent studies in small animal models focused on the engineering of nerve conduits by using natural (biological conduits) or artificial (synthetic conduits) materials. Nerve conduits serve as a bridge between the proximal and distal stump, providing a scaffold upon which cells can migrate between the two nerve stumps. The most significant advantage of a nerve conduit is its ability to create an ideal microenvironment for neuronal recovery and nerve growth, especially for complex defects.^12^ Moreover, nerve conduits make the repair site less susceptible to perineural fibrosis and infiltration by inflammatory cells. For all these reasons, an ideal nerve conduit should have properties like porous, flexible, thin, biocompatibility, permeability, flexibility, biodegradability, neuroinductivity, and neuroconductivity with an appropriate surface.^13^ To date, in order to avoid the possibility of external body reaction, scar formation and inflammation of neighboring tissues, the choice of material for nerve conduits and scaffolds has shifted toward the more biocompatible, biodegradable, and bioresorbable synthetic polymers such as polyglycolic acid (PGA), poly‐lactidecaprolactone (PLCL), polycaprolactone (PCL), and recently also polyurethane, which induce only minimal foreign body reaction and excellent nerve regeneration.^14^, ^15^ In other case, myelination and collagen IV deposition were also observed.^16^

Collagen based scaffold also shown to be effective in nerve repairing, in rat model and in humans as FDA‐approved materials.^17^, ^18^

New biomaterial processing techniques, such as electrospinning or bioprinting, allow the development of special neural guides, designed to simulate the structure of the extracellular matrix, increase the contact surface for the regenerated axon and further stimulate its growth. Currently, electrospinning technique is available to produce degradable artificial nerve conduits with aligned or random nanotopographies.^19^, ^20^ In particular, as reported in several studies, aligned polymer fiber‐based constructs present sub‐micron scale structure. This characteristic improve peripheral nerve repair by promoting Schwann cell migration.^21^

Poly(1,4‐butylene succinate) (PBS) is another example of water insoluble biopolymer synthesized by the polycondensation of 1,4‐butandiol with succinic acid. Given its chemical structure, PBS shows excellent melt processability, a proven biocompatibility and biodegradability,^22^ and a good versatility when employed as material for various biomedical applications. Its versatility includes application in bone regeneration or myocardial tissue replacement and different manufacturing approaches, including salt leaching, electrospinning or extrusion techniques.^23^, ^24^, ^25^, ^26^, ^27^, ^28^ In this study, PBS was tested as biomaterials for the production of nanostructured conduits for severed nerve regeneration. To this aim, microfibrillar PBS‐based 3D scaffolds, produced by electrospinning technique,^29^ were implanted and tested for stimulating and guiding peripheral nerve functional regeneration in rat models of sciatic nerve transection, in order to assess their in‐vivo biocompatibility and effectiveness as nerve guidance structures and improve regeneration. The promising obtained results encourage to further investigate the use of this innovative and efficient surgical strategy for treatment and bridging of extreme nerve injuries, proposing PBS‐based microfibrillar 3D scaffolds as regenerative sheath and in situ therapeutic reservoir for the biological stimulating factors that naturally improve axonal reconstruction and accelerate overall functional recovery.

## 2 MATERIALS AND METHODS

### 2.1 Poly‐butylene succinate (PBS) scaffolds fabrication by electrospinning technique

Poly(1,4‐butylene succinate), extended with 1,6‐diisocyanatohexane (PBS, Sigma‐Aldrich, UK), was dissolved in dichloromethane (15% w/v) obtaining a clear polymer solution. Of note, 30 ml of this solution was used to prepare each batch. The electrospinning process was carried out horizontally with 15 kV voltage (Spellman CZE 1000 R) and a constant polymeric solution rate of 0.8 ml/min obtained through a programmable syringe pump (Aitecs PLUS SEP‐21. The electrospun scaffold was collected on an aluminum foil wrapped around an earthed rotating collector 15–20 cm away from the tip of the needle.

### 2.2 PBS scaffolds characterization by scanning electron microscopy (SEM) and microcomputed tomography (μCT)

Morphological characteristics of scaffolds were investigated with a scanning electron microscope (ESEM Philips XL30) operating at 5 kV. Each sample was deposited onto a carbon‐coated steel stub, dried under vacuum (0.1 Torr), and sputter‐coated with gold (15 nm thickness) prior to microscopy examination.

3D structure of the scaffold was analyzed by using a μCT scanner (Skyscan 1272, Bruker Kontich, Belgium) at a source voltage and a current of 40 kV and 250 mA respectively, with a total rotation of 180° and a rotation step of 0.3°. No filter mode was chosen for the acquisitions. The image pixel size was 2.6 μm and the scan duration was about 3 hr for every sample. The scanning dataset obtained after the acquisition step consisted of images in 16‐bit tiff format (3238 × 4904 pixels). The 3D reconstructions were carried out using the software NRecon (version 1.6.10.2) starting from the acquired projection images. The obtained 2D‐images had color depth of 8 bit with 265 grey levels. After that, the whole set of raw images were displayed in a 3D space by the software CTVox.

### 2.3 Animals

All experiments described within this study were performed in the Istituto Zooprofilattico Sperimentale della Sicilia “A. Mirri” (Palermo, Italy) and authorized by the Ministry of Health (Rome, Italy; Authorization Number 456/2018‐PR). Procedures involving animals were carried out in accordance with the Italian Legislative Decree N° 26/2014 and the European Directive 2010/63/UE. Twenty adults' male Wistar rats weighting between 150 and 200 g (Charles River Laboratories, Calco, Italy) were used for this study. Animals were housed two per polypropylene cage and kept in controlled temperature (22 ± 2°C), humidity (50–55%) and light (12 hr light/dark cycle), with access to food and water ad libitum. Rats were allowed to acclimate for 2 days prior to experiments.

Rats were randomly divided into two experimental groups. In Group 1 (G1; *Control*; *n* = 10), sciatic nerves were transected and repaired with standard epineural microsurgical sutures (simple primary repair). In Group 2 (G2; *Nanofiber wrap*; *n* = 10), a PBS‐based scaffold was implanted following neurotmesis at the severed nerve stumps without epineural repair. The outcomes were evaluated by electrophysiological assessment, magnetic resonance images (MRI), muscle atrophy evaluation and histological analysis after two post‐surgery survival periods of 30 and 120 days, respectively.

### 2.4 Surgical procedure and scaffold implantation

Surgical procedures were performed under aseptic conditions using a power focus surgical microscope (Carl Zeiss, Germany). Animals were induced to anesthetic depth with inhaled isoflurane at 2% and then anesthetized with intramuscular (i.m.) injection of Zoletil(r) (tiletamine/zolazepam; 10 mg/kg) and Domitor(r) (medetomidine hydrochloride; 0.5 mg/kg).^30^ All rats were operated by the same surgeon and only on a limb, so that mobility, self‐sufficiency in eating and drinking were allowed. Before surgery, the hair was clipped over the thigh and surgical area was scrubbed with a 70% alcohol solution. A small skin incision of 40 mm was created in the right limb of each rat over the gluteal muscle along the femoral axis. With a muscle‐splitting approach, that is the biceps femoris and superficial gluteal muscles were detached with blunt dissection, the sciatic nerve located 4 mm below the skin was exposed and then sharply transected at the mid‐thigh level, proximal to the tibial and peroneal bifurcation, using microscissors. After transection, a 7 mm long nerve gap was created only in the nanofiber wrap group (G2) injured nerves, resulting from a “facilitated” nerve retraction. In the control group (G1), the proximal and distal nerve stumps of the injured nerve were sutured using three 6/0 monofilament nylon epineural sutures (Ethicon). In the experimental group G2, the proximal and distal nerve ends (included the interstump gap) were wrapped with the PBS nanofiber scaffold (12 × 12 mm) to surround the whole repair site, with no primary repair. The 12 mm long polymeric wrap enabled a 7 mm nerve gap when used as guidance tube, due to the 2.5 mm overlap needed on each end of the severed nerve. The wrap was 0.5–1 mm larger than the nerve diameter. The sciatic nerve was kept moist with sterile saline solution throughout the surgical procedure. In all groups, muscle wound beds were sutured with 2/0 Vicryl. The incised skin was closed with surgical staples with 6–7 sutures and disinfected with povidone‐iodine (Betadine) solution. I.m. atipamezole (Antisedan) (300 μg/kg) was used in order to awaken all rats. Carprofen analgesia (5 mg/kg) and Enrofloxacin (5 mg/kg) were daily administered for 1 week to each rat immediately after surgery to prevent infection. Animals were then transferred and housed one per cage and given an identification number. They were monitored on a daily basis for infection, self‐mutilation, and signs of distress. Subsequent postoperative observations and procedures were performed at 30 and 120 days respectively.

### 2.5 In‐vivo magnetic resonance imaging (MRI) measurements

Rats were anesthetized with isoflurane and imaged by a Bruker 7‐T MRI instrument (Germany) at 30 and 120 days after implantation (*n* = 5 per group). The parameters for T2 weighted sequence were: gradient echo with TR/TE/flip angle: 250 ms/33 ms/15 ms and matrix pixel 256 × 256. Images could be taken from the sagittal and axial directions to observe in connection with the regenerated nerve.

### 2.6 Electrophysiological assessment

To test the restoration of functionality of the regenerated nerve through the implant, electrophysiological recordings were performed at 120 days post‐surgery (*n* = 4 per group), before the animals were sacrificed for histological analysis and muscles dissection, by means of motor unit number estimation (MUNE). MUNE is a non‐invasive electrophysiologic technique, originally described by McComas and co‐workers over three decades ago, that has been used to monitor the functional status of a motor unit pool in vivo and to estimate the number of functioning motor neurons innervating the muscles being tested.^31^ This method is based on compound muscle action potential (CMAP) response that represents the electrophysiological output from a muscle or group of muscles following supramaximal stimulation of a peripheral nerve. MUNE was performed on all animals according to an adapted version of Gordon and co‐workers.^32^ Briefly, rats were anesthetized as described in detail previously and placed in the supine position. Surface temperature at 37°C was maintained with a thermostatic warming plate to avoid hypothermia. Animals were fixed with tape on a smooth table to prevent movement artifacts due to the electrical stimulation, the lower limbs gently stretched and spread forming an approximately 45° angle to the spine, the sciatic nerve was then stimulated by using a device with two mono polar needle electrode that were inserted subcutaneously at the root of the hind limb. The muscular response to the electrical nerve stimulation was recorded with a pair of monopolar recording needle electrodes placed onto the belly and onto the tendon of the *tibialis anterior* (*TA*) and the *gastrocnemious* (*GA*) *medialis* muscles, respectively. Once the optimal position was found, as assessed by evoked CMAP on both muscles, the stimulating electrode was kept in a constant position by means of a mechanical apparatus. Sciatic nerve stimulation was performed with square‐wave pulses of 0.1 ms duration with gradually increasing stimulus intensity until the first reproducible, “all‐or‐none” S‐MUAP (surface‐detected motor unit action potential) was evoked. A collection of 15 reproducible S‐MUAPs for each muscle was recorded by stimulation of the nerve. A supra‐maximal stimulation (at 10% above the threshold) was then performed in order to evoke the maximum CMAP. The MUNE was then calculated using the following equation:

MUNE=Peak to peak amplitude of the maximum CMAP/Peak to peak amplitude of the averageS−MUAP.

MUNE was obtained both for operated and for contralateral limbs in control (*n* = 4) and nanofiber wrap (*n* = 4) groups. The final data were expressed as mean ± *SD*.

### 2.7 Muscle atrophy evaluation by weight ratio

At the time of sacrifice, 30‐ and 120‐days post‐surgery (*n* = 5 per group), *gastrocnemius* (*GA*) and *TA* muscles were carefully dissected out, on both sides (right R, operated side; left L, controlateral side), dividing the tendinous origin and insertion from the bone. Then, samples were harvested in their entirety and weighed for comparative analysis of their mass. The reduction in muscle mass was assessed by calculating the ratio of muscle weight between the two limbs (R/L), both for GA and TA muscles, using the following formula: MWR = weight of operated muscle/weight of controlateral muscle.

### 2.8 Histological analysis

Rats (*n* = 10 per group) were sacrificed under general anesthesia, after a post‐surgery time of 30 and 120 days for histological analysis. The skin and superficial and deep hind limb muscles were dissected under a surgical microscope, and 10 mm of sciatic nerve (distal to the site of nerve lesion) was removed from each animal of G1 and G2 at the same anatomic location (5 mm distal to where the sciatic nerve crosses the tendon of the internal obturator muscle). Both sciatic nerves were harvested: the normal left side (used as healthy control) and the right one (surgery). Nerve samples were immediately fixed in 4% paraformaldehyde in phosphate‐buffered saline (PBS) for 2–4 hr and then washed and stored in 0.2% glycine in PBS. The specimens were first washed with PBS and postfixed with 2% osmium tetroxide for 2 hr, washed with 3–5 passages in distilled water, dehydrated with an increasing alcohol series, embedded in paraffin, and finally cut into transverse thin sections (3–5 μm thick) and stained with hematoxylin and eosin for morphometric analyses.^33^ One more sciatic nerve aliquot (5 mm proximal to the site of nerve lesion) was directly stained with hematoxylin and eosin for histological evaluation. Slides were evaluated by an observer blinded (RP) to the experimental groups for overall nerve architecture, quantity of regenerated nerve fibers and Wallerian degeneration. All nerve sections were evaluated under optical microscope (Leica DMR, Germany) and photographed with a high‐resolution camera (Nikon DS‐Fi1, Japan). The sciatic nerve area was calculated for each experimental group (G1 and G2) at 30 and 120 days post‐surgery. Six random microscopic fields per nerve were captured at 1000x magnification and evaluated with image analysis software (Image J), based on gray and white scales. Myelinated fibers were semiautomatically recognized by the software and the remaining fibers were manually redrawn. Total fiber number (N) was estimated by measuring sciatic nerve area and area of sample at ×1000 magnification and multiplying by the number of fibers in sample; fiber density (FD = *N*/mm^2^) is calculated by dividing the number of fibers within the sampling field by its area.^34^ All values of morphometric parameters were expressed as mean ± *SD*.

### 2.9 Statistical analysis

All experiments were performed in triplicate collecting for each experiment a number of samples *n* = 6, and values were expressed as mean ± standard deviation. ANOVA followed by a Tukey post hoc test was performed using the QI Macros SPC Software for Excel to determine the significance of results. *p*‐value < .05 was defined as the level of statistical significance.

## 3 RESULTS

### 3.1 Fabrication and characterization of PBS scaffolds

**FIGURE 1:**
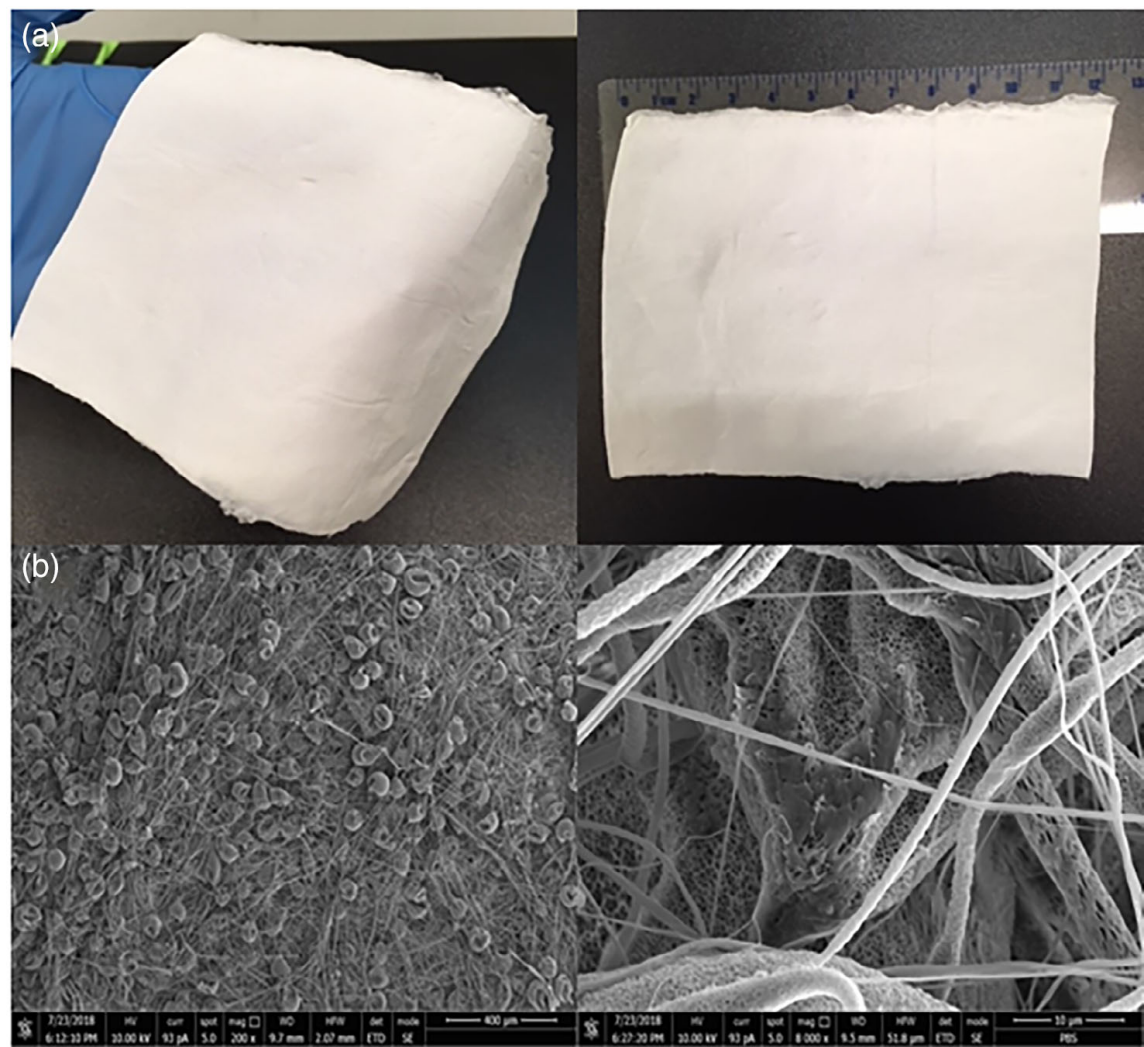
Photographs of a polybutylene succinate (PBS) scaffold prepared by electrospinning (Panel a). SEM images of polybutylene succinate scaffold at magnification 200 and ×8,000 (Panel b)

The electrospinning procedure used for the production of PBS scaffolds tested in this study was already explored in a previously published work.^29^ For this study, the polymeric solution extrusion rate was increased from 0.6 to 0.8 ml/min, in order to improve the density of the final electrospun scaffold and the mechanical resistance to the surgical procedure. Scaffold were produced as flexible thin sheet (9 × 12 cm), with an average thickness of 0.5 mm, as shown in the photograph of Figure 1 (Panel a).

**FIGURE 2:**
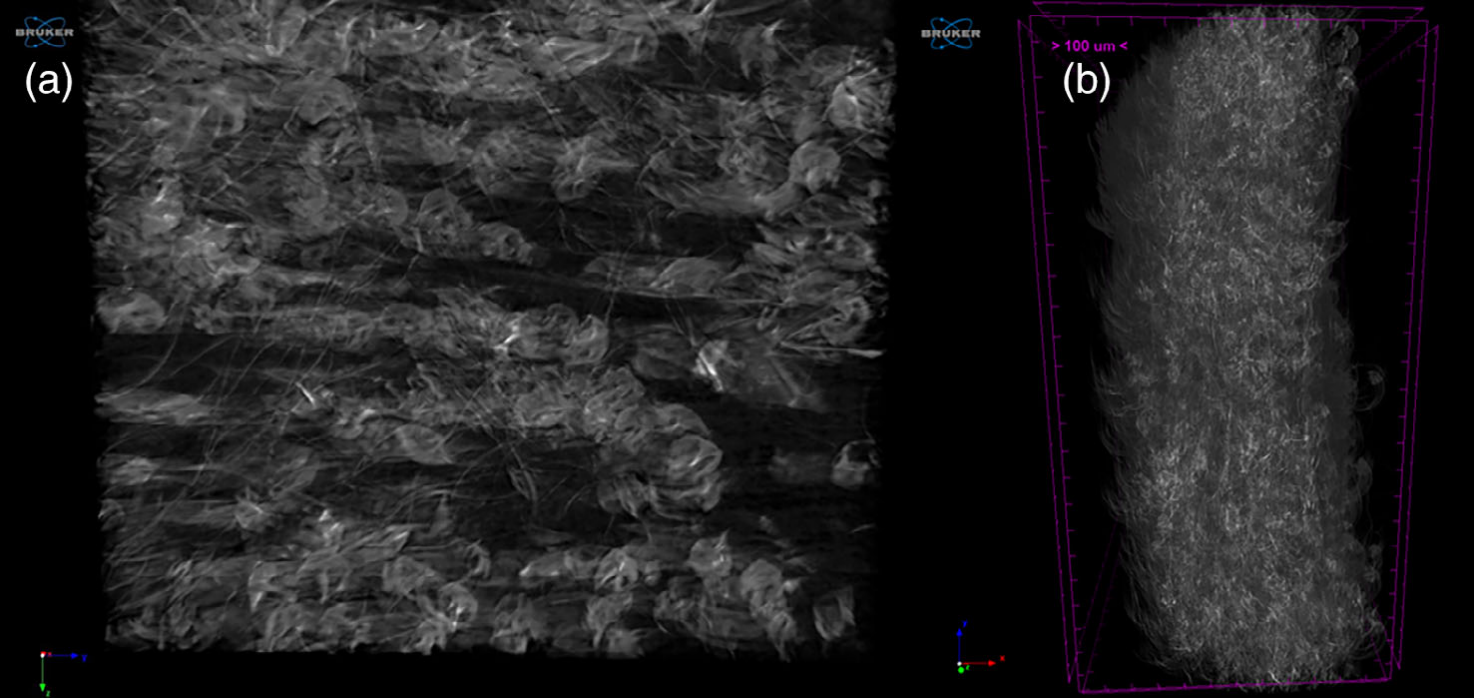
3D reconstruction obtained by μCT analysis evidencing the microfibrillar structure (Panel a) and the thickness of the scaffold (Panel b)

After production, PBS scaffold were analyzed by SEM in order to highlight the microscopic features, porosity, and micro‐fibrillar structure. As shown in SEM images, (Figure 1, Panel b), the inner structure of the scaffold is highly porous. Interestingly, the microstructure of the scaffold shows microfibers with a diameter between 1–5 μm, alternating with the presence of collapsed‐balloons like structures along the microfibers. This finding may be related to the increased polymeric solution extrusion rate from 0.6 to 0.8 ml/min, during electrospinning process. Moreover, at higher magnification, a superficial microporosity is observable in the scaffold forming fibers. Interestingly, the 3D reconstruction obtained by μCT analysis evidenced the microfibrillar structure also in the internal part of the scaffold and an adequate porosity of the materials (Figure 2a). This analysis was also used to measure the exact thickness of the scaffold that resulted about 300 μm (Figure 2b).

### 3.2 Evaluation of nerve regeneration

#### 3.2.1 MRI of nerve repaired with PBS wraps

The 7 Tesla preclinical MRI tomography allowed to qualitative predict the absence of strong inflammatory reaction and any anomalies at a morpho‐structural level, consistent with regenerative process of the sciatic nerve. Actually, the analysis of MRI images was focused in evaluating changes in signal intensity, in particular on T2‐weighted images, in order to identify potential anomalies in the cross‐sectional area and nerve course, as well as disorganization or absence of the typical fascicular pattern. Interestingly, the MRI scan of the region of the hind limbs, left and right, of the animal showed an improvement of the regenerative process from 30 to 120 days in the G2 group.

**FIGURE 3:**
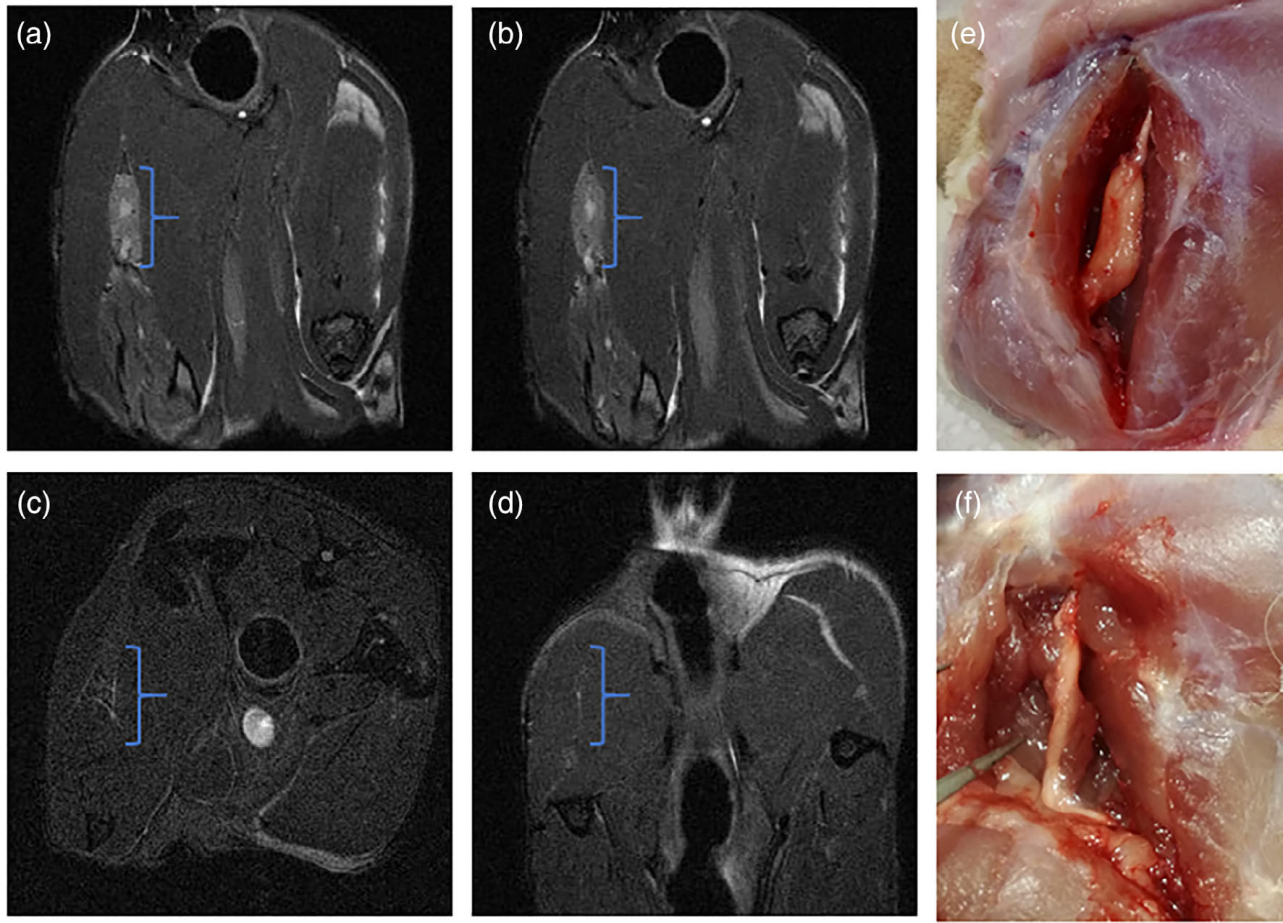
(a, b) MRI scans (hind limbs, left and right) of the Group 2 (as example were reported the images of two animals) after 30‐days post implant. The surgery (e) clearly show portion of the scaffold (as example was reported the image relate to the animal a), perfectly adapted to the sciatic nerve segment under consideration. (c, d) MRI scans of the G2 (as example were reported the images of two animals) after 120‐days post implant. The surgery (f) clearly show the reabsorption of the scaffold and generation of normal nerve (as example was reported the image relate to the animal c)

In particular, at 30 days post implant, it is possible to highlight a hyperintense signal in T2 (Figure 3a,b,e) in the right limb, expected when an inflammatory process occurs; it is also possible to view the presence of the tubular scaffold. The portion of the scaffold, with the severed nerve inside, is located between the GA muscle, the soleus muscle, and the cranial tibialis muscle.

Differently, the MRI scanning analysis after 120 days post implant, showed the reduction of the hyperintense signal in T2, which is coherent with absence of inflammation process, and almost the total reabsorption of the scaffold (Figure 3c,d,f).

#### 3.2.2 Electrophysiological findings

Sciatic nerve functional recovery was estimated by performing electrophysiological analysis only at 120 days postoperatively, before the animals were sacrificed, as reinnervation phenomena need several weeks to months to be seen.^35^ The number of estimated motor units (MUNE) was calculated both for operated (right) and for healthy contralateral (left) limbs in control (*n* = 4) and nanofiber wrap (*n* = 4) groups, for GA and TA muscles (TA). T‐Test analysis for independent variables was performed on MUNE of each muscle (GA and TA) with the “side” variable (right vs. left) as intra‐subject factor. As regards the GA muscle, a significant difference only in the G2 (nanofiber wrap group) was found (mean right: 27.25 ± 6.42; mean left: 56.02 ± 6.79; *p* < .05). No statistical significance (*p* > .05) has been observed in control group (G1) between the GA muscles of both sides. We achieved opposite results for TA muscle: the mean MUNE for the operated side muscle was significantly lower than that of the normal contralateral side only in control group (G1) (mean right: 13.34 +/− 2.65; mean left: 77.12 +/− 25.38; *p* < .05); while in PBS scaffold group (G2), it was not significantly different in comparison with healthy limb muscle (*p* > .05). Box and whiskers plot of significant electrophysiological data are shown in Figure S1.

#### 3.2.3 Muscles atrophy findings: Ratio of the masses

Muscle weight ratio (MWR) provided a gross estimate of target muscle atrophy (muscle mass reduction). Therefore, the *GA* and the *TA* muscles of both operated and healthy limbs were explanted at 30 and 120 days and weighed (Figure S2). The collected average weights were used to calculate the Gastrocnemius Muscles Weight Ratio (GAMWR) and Tibialis Anterior Muscles Weight Ratio (TAMWR). Actually, a general increase in weight of all tested subjects was expected during evaluation period, due to normal growth, not being animals on a restricted diet regime. Results are reported in Table 1.

**TABLE 1:** Summary of muscle weight ratio (MWR) of the *gastrocnemius* (GAMWR) and the *tibialis anterior* (TAMWR) muscles of both operated and healthy limbs explanted at 30 and 120 days, calculated from GA and TA weights

	30 Days				120 Days			
	G1 (control)		G2 (scaffold)		G1 (control)		G2 (scaffold)	
	Operated limb (gr)	Healthy limb (gr)	Operated limb (gr)	Healthy limb (gr)	Operated limb (gr)	Healthy limb (gr)	Operated limb (gr)	Healthy limb (gr)
GA	1.008 (0.41–1.38)	1.712 (1.06–2.2)	0.93 (0.33–1.32)	1.768 (1.01–2.5)	0.7620 (0.59–0.86)	1.216 (1.01–1.43)	0.6460 (0.31–0.84)	1.2320 (0.92–1.59)
GAMWR	0.6041 (0.262–0.905)		0.5027 (0.326–0.60)		0.6368 (0.495–0.851)		0.5253 (0.336–0.762)	
T‐TEST	*t* = 0.8874; *df* = 8; *p* = .40				*t* = 1.1235; *df* = 8; *p* = .29			
TA	0.63 (0.35–1.08)	1.118 (0.67–1.48)	0.6160 (0.11–1.11)	1.2420 (0.55–1.99)	0.3720 (0.31–0.46)	0.71 (0.55–0.78)	0.2480 (0.15–0.37)	0.56 (0.45–0.66)
TAMWR	0.5519 (0.357–0.729)		0.4534 (0.2–0.609)		0.5316 (0.397–0.63)		0.4458 (0.28–0.60)	
T‐TEST	*t* = 1.0037; *df* = 8; *p* = .34				*t* = 1.1142; *df* = 8; *p* = .29			

#### 3.2.4 Histological findings: Counting of regenerated fibers

**FIGURE 4:**
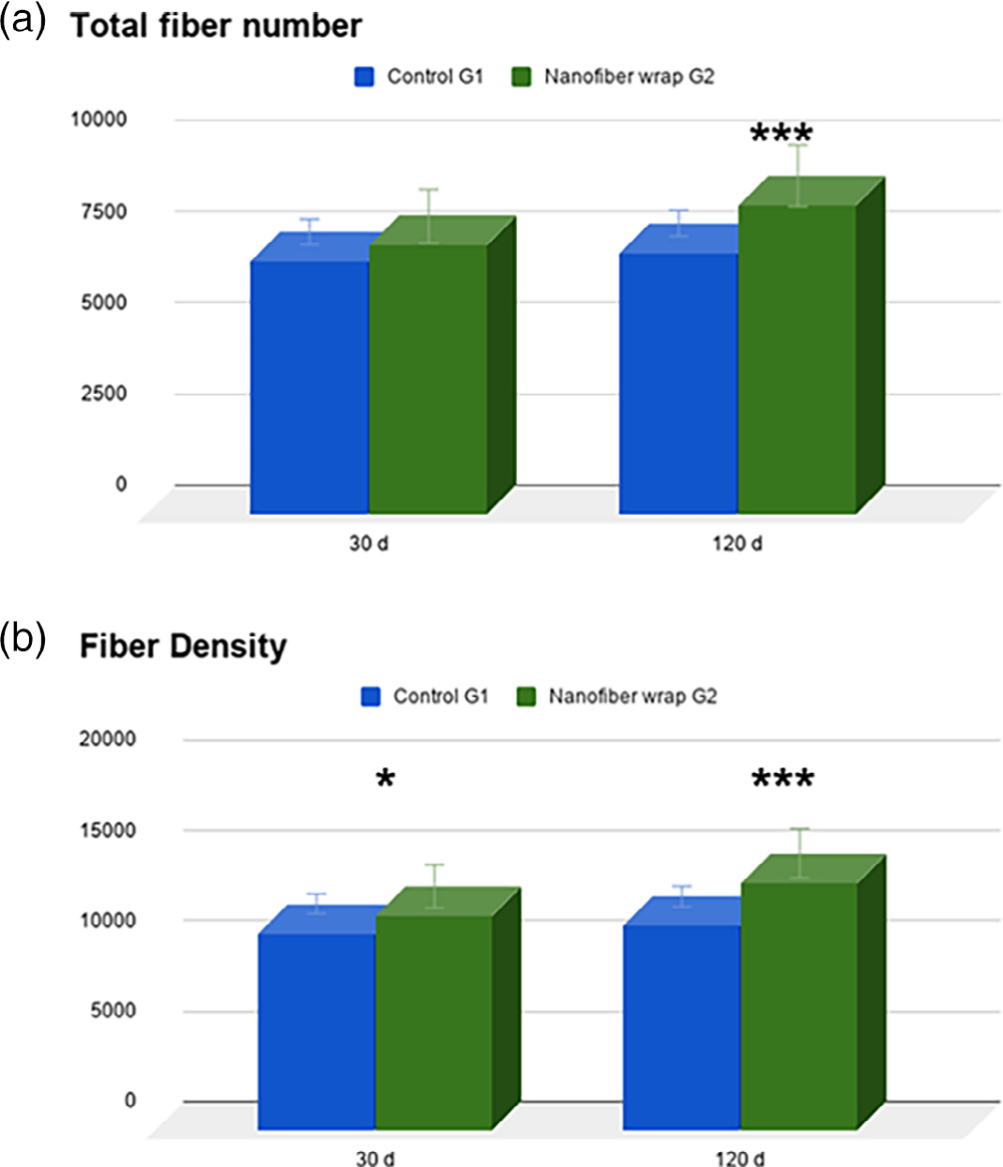
(a) Total fiber number after 30‐ and 120‐days post‐surgery. *** *p* < .001. (b) Nerve fiber density after 30‐ and 120‐days post‐surgery. * *p* < .05; *** *p* < .0001. Sample area at ×1000 magnification is 5.639 μm^2^

The analysis of the normal sciatic nerve with HE staining allowed us to observe the typical undulated and parallel organization of the nerve fibers (Figure S3). After 30 and 120 days, the total fiber number (Figure 4a) and fiber density (Figure 4b) at the site of the nerve transection were evaluated. After 30 days, control group G1 showed 6940 ± 296.64 total fiber number; in G2 were 7368 ± 801.31. The total fiber numbers (Figure 4a) in the G2 was found not statistically different from the G1 contralateral control. Differently, at 120 days post‐surgery the nanofiber wrap group showed greater number of fibers than G1, being statistically different from control group value (G1: 7176 ± 180.53; G2:8476 ± 765.68).

The nerve fiber density (fiber number/mm^2^) was statistically different for G1 and G2 group both at 30‐ and 120‐days post‐surgery (Figure 4b). In normal sciatic nerve, the fiber density was 10,936 ± 342.51 fibers/mm^2^ at 30 days, and 11,335 ± 415.12 fibers/mm^2^ at 120 days; while there were increase in G2 nanofiber wrap group (11,899 ± 829.22 fibers/mm^2^ at 30 days, and 13,737 ± 940.43 fibers/mm^2^ at 120 days. Data of cross sectional area are reported in Table S1.

## 4 DISCUSSION

The use of planar microfibrillar scaffold implanted as conduit in the attempt to repair severed peripheral nerves proved to be a potential efficient surgical technique to improve the regeneration of extreme nerve injuries. Actually, this strategy resulted easier than other surgical methods used in the past (end‐to‐end sutures, transplant, osteotomies, etc.). For this aim, PBS based electrospun planar scaffold, shown to be promising candidate as implantable 3D biomaterial for stimulating and guiding peripheral nerve functional regeneration in rat models of sciatic nerve transection and reduce the time for a complete nerve regeneration, if compared with already studied nerve guide conduit, tested on sciatic nerve in rat, also without the releasing of previously loaded growth factor or neuroprotective agent.^14^, ^15^ In the present study the biocompatibility and biodegradability of the PBS‐based scaffold was proved, with no physiological complications or rejection of the device and a complete reabsorption in 120 days post implant was find by NMR image analysis.

**FIGURE 5:**
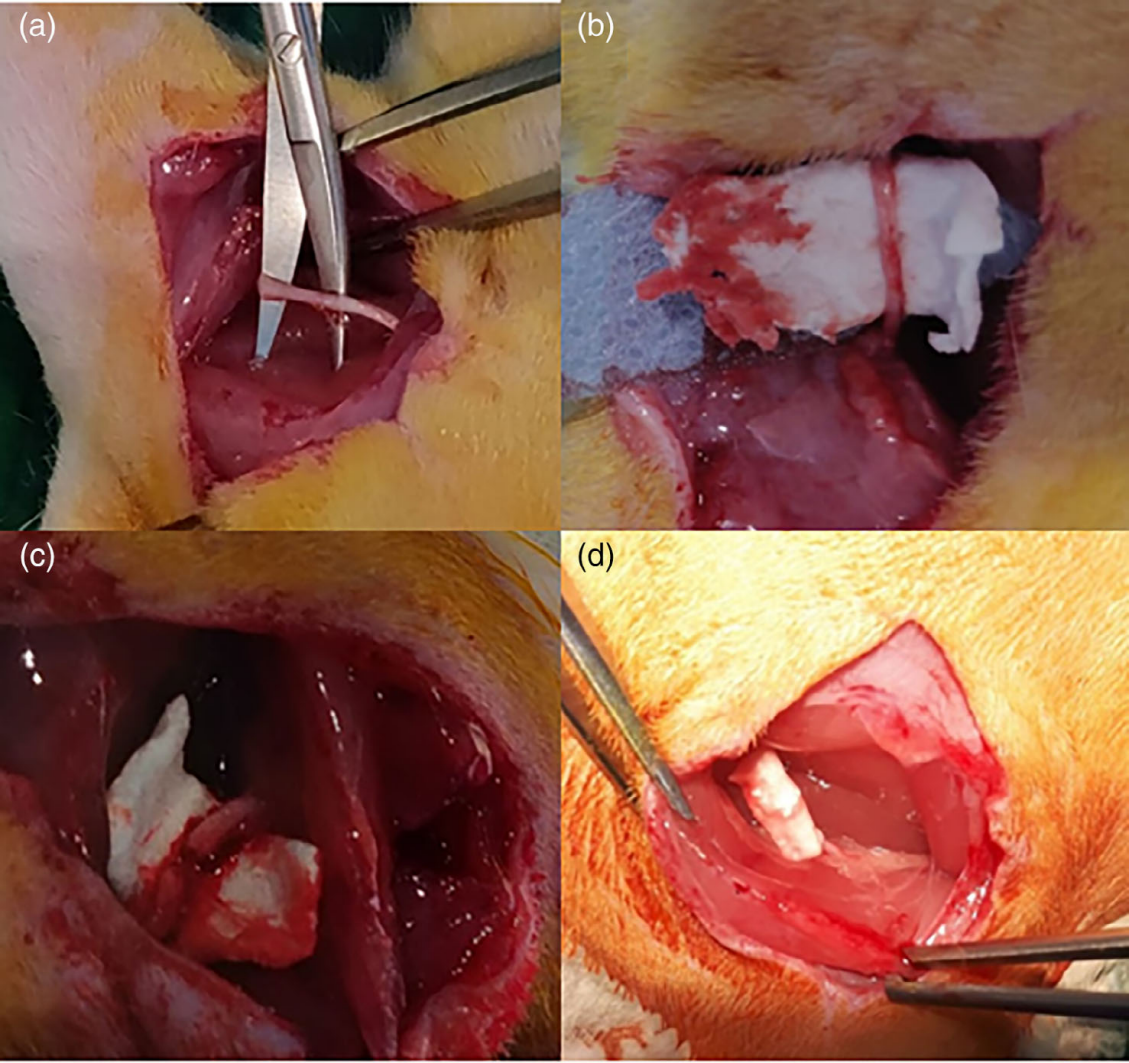
Surgical steps of polybutylene succinate planar scaffold implantation: (a) nerve isolation; (b) insertion of the planar scaffold; (c) nerve section; and (d) nerve wrapping

Specifically, two animal populations with overlapping surgical nerve procedure were compared: the control group treated with a proximal and distal epi‐perineural suture of the severed sciatic nerve, as the most used and reliable surgical technique nowadays; and a pioneering group with the severed sciatic nerve wrapped with the PBS nanofiber scaffold (12 mm long) to surround the whole repair site, with no primary repair (Figure 5). In this second group, the 12 mm long polymeric wrap maintained a 7 mm nerve gap between each end of the severed nerve, and was used as guidance tube between nerve flaps.

The demonstration of the nerve regeneration was based on the evaluation of electroneurography, the weight of muscles, and histological examination of regenerated nerves and locomotor activity of animals, representing the current gold analytical standard for similar case studies.

In principle, MRI at 7 T allowed to evaluate possible anomalies at a morphological and structural level in both the inflammatory and regenerative processes. It was given particular attention to changes in signal intensity in T2 images, in cross sections and in the course of the nerve, as well as in pattern of disorganization or in the eventual absence of the typical collate pattern. At 30 days due to inflammation, it was found a hyper intense signal in T2 and the presence of tubular scaffold. The comparison at 120 days between the two groups has shown the quite complete absence of inflammation and the almost certain reabsorption of the scaffold that confirmed the biocompatibility of PBS.

As expected following nerve transections, neurophysiological findings showed a significant reduction of MUNEs in operated limb when compared to contralateral uninjured nerve.^36^ However, this difference was limited to *GA* muscle in G2 and to the *TA* muscle in G1. It was observed that even proximal sciatic neuropathy preferentially affect peroneal fibers, leading to worse deficit in TA muscle. This finding in G1 is coherent with the literature.^35^ Conversely, we found better recovery in TA muscle in the G2 (PBS scaffold group); while we strongly believe that the limited number of subjects enrolled severely affects the results, and only qualitative considerations can be made, this could suggest a specific effect of PBS scaffold in guiding peroneal fibers. If confirmed in a larger series this data can represent a basis for using scaffolds when peroneal fibers are more severely injured.

Actually, muscles mass indexes of *gastrocnemius* muscle (GMWR) and *tibialis anterior* (TAMWR) supported the tropism of muscles innervated by the chosen nerve. A denervated muscle undergoes atrophy, with a speed proportional to the muscle mass and denervation. The re‐innervation of the surviving fibers, within certain limits, causes the interruption of the degenerative phenomenon and progressively the muscle should recover its tropism. On this basis, the weight of the studied muscles, innervated by branches of the sciatic nerve, represented an indirect index in the evaluation of the nerve regeneration. The statistical analysis of the GMWR with the T‐test showed no statistically significant difference between G1 and G2 either at 30 days or 120 days after surgery (30 days: *p* = .4008; 120 days: *p* = .2938). In addition, the corresponding analysis of the TAMWR showed similar results as well (30 days: *p* = .3449; 120 days: *p* = .2975).

The above findings are certainly related to a normal gait regained by the animal as early as 30 days after the PBS implant.

Histological analysis of the nerves provided two further parameters supporting the positive advantages of PBS scaffolds in nerve regeneration: the number of fibers and the density of the fibers. Given that the ability to synthesize proteins is largely owned by the neurosome, the distal segment quickly loses its ability to transmit action potentials and undergoes a series of degenerative changes called Wallerian degeneration. Otherwise, the proximal stump generates axonal gems that, organized in growth cones, will try to reach the target organs for re‐innervation. This process can take place only if the continuity between the proximal and distal stump is maintained. This explains why surgical repair is always necessary. Finally, the observation of locomotor activity, despite an altered semi‐erect position, revealed a progressive normalization of the posterior locomotor system movements (legs movement did not appear impaired) just starting from 30 days post implant.

## 5 CONCLUSIONS

The present study demonstrates that the use of the planar PBS scaffold is a more effective method of fixing the injured two portions (proximal and distal) of the sciatic nerve, to preserve nerve continuity and promote its regeneration. The interpretation of the difference obtained by electroneurography, the weight of *GA* and *TA* muscles, histological examination of regenerated nerves and locomotor activity of animals leaves no doubt that there is a real improvement in the regeneration process of the sciatic nerve, used here as a nerve model, if the animals treated with the scaffold are compared with those in which the severed nerve has been sutured with a traditional technique.

The results demonstrated that there is an important nerve regeneration action due to both mechanical and vehicle support of the scaffold and in the same way an adequate biodegradability, as observed from high resolution MRI investigation, that highlight the potentiality of PBS as biomaterial for nerve regeneration.

The results obtained encourage new research perspectives aimed at testing the use of a three‐dimensional structure such as the PBS planar scaffold, on a larger nerve sample model, to subsequently promote its use in clinical practice, considering an advancement of the standard surgical technique and the advantage of a reduction in clinical healing times and therefore also in costs.

## CONFLICT OF INTEREST

The authors indicated no conflict of interests.

## Supporting information


**Appendix** **S1.** Supporting Information.

## Data Availability

The data that support the findings of this study are available from the corresponding author upon reasonable request.
