# Supplementary material for: Polybutylene succinate artificial scaffold for peripheral nerve regeneration
Source: J Biomed Mater Res B Appl Biomater. 2021 Jun 27;110(1):125–34. doi: 10.1002/jbm.b.34896 (PMC9290626; doi:10.1002/jbm.b.34896)
Supplement: Supplementary file 1 — Appendix S1. Supporting Information. [file JBM-110-125-s001.docx]

**Supplementary informations**

**Polybutylene succinate artificial scaffold for peripheral neuronal regeneration**

**Luca Cicero^1^, Mariano Licciardi^2^*, Roberta Cirincione^1^, Roberto Puleio^1^, Gaetano Giammona^2^, Giuseppe Giglia^3^, Pierangelo Sardo^3^, Giulio Vigni^4^, Alessio Cioffi^4^, Antonino Sanfilippo^4^, Giovanni Cassata^1^**

^1^Istituto Zooprofilattico Sperimentale della Sicilia “A. Mirri”, Via Gino Marinuzzi 3, 90129 Palermo, Italy.

^2^Dipartimento di Scienze e Tecnologie Biologiche Chimiche e Farmaceutiche (STEBICEF), Università degli Studi di Palermo, 90128 Palermo, Italy.

^3^Dipartimento di Biomedicina, Neuroscienze e Diagnostica Avanzata (BiND) Università degli Studi di Palermo, 90123 Palermo, Italy.

^4^Dipartimento di Discipline Chirurgiche, Oncologiche e Stomatologiche, Università degli Studi di Palermo, 90127 Palermo, Italy.

*Corresponding author: [mariano.licciardi@unipa.it](mailto:mariano.licciardi@unipa.it)


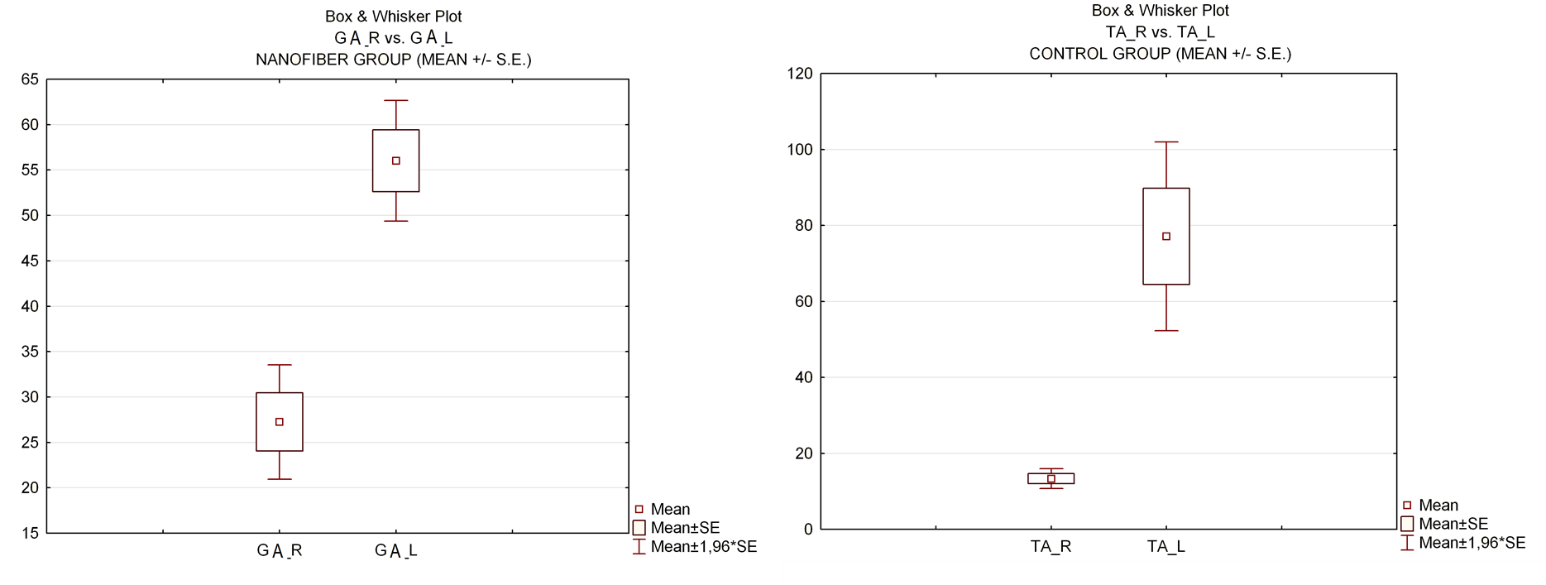


Figure 1S: Box and Whiskers plot of significant electrophysiological data recorded on gastrocnemius (GA) and tibialis anterior (TA) muscles with the "side" variable, right (R) vs. left (L), method. For the analyzed samples, right (R) vs. left (L), P is always < 0.05.


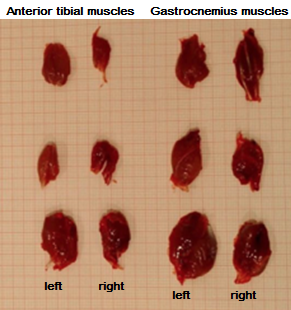


Figure 2S: Tibialis anterior and gastrocnemius muscles of left and right limbs respectively, withdrawn from G2 (*Nanofiber wrap*) 120 days post scaffold implant.


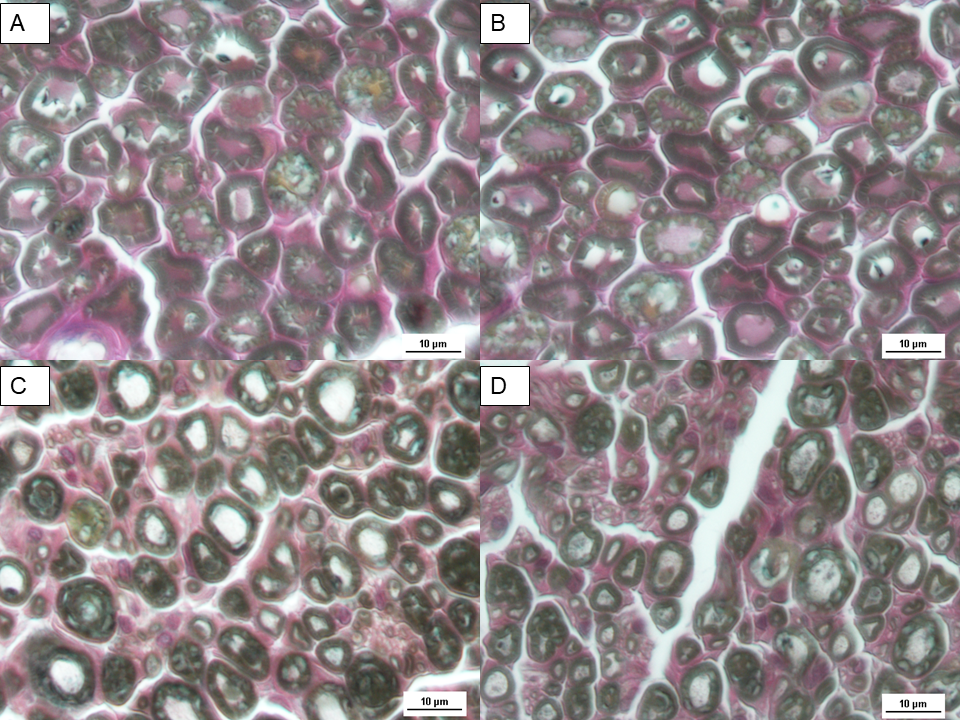


Figure 3S: Histology images of G1 at 30 (A) and 120 (B) days post-surgery. G2 at 30 (C) and 120 (D) days post-surgery. Osmium Tetroxide fixation and Hematoxylin-eosin stain. Scale bar 10 µm.

Table 1S: Cross sectional sciatic nerve area of G1 and G2 at 30 and 120 days post-surgery.

| **CROSS SECTIONAL AREA** | | | | |
| --- | --- | --- | --- | --- |
|  | **G1** | | **G2** | |
|  | **30 days** | **120 days** | **30 days** | **120 days** |
| Media | 632.695 µm^2^ | 626.741 µm^2^ | 607.285 µm^2^ | 590.1365 µm^2^ |
| SD | 28.223 µm^2^ | 23.831 µm^2^ | 41.603 µm^2^ | 55.526 µm^2^ |
